# A Green Integrated Approach to Multifunctional Silver Nanoparticles Derived from *Aronia melanocarpa*

**DOI:** 10.3390/pharmaceutics17050669

**Published:** 2025-05-20

**Authors:** Andreia Corciova, Cornelia Mircea, Adrian Fifere, Ioana-Andreea Turin Moleavin, Ana Flavia Burlec, Bianca Ivanescu, Ana-Maria Vlase, Monica Hancianu, Irina Macovei

**Affiliations:** 1 Faculty of Pharmacy, “Grigore T. Popa” University of Medicine and Pharmacy, 16 University Street, 700115 Iasi, Romania; maria.corciova@umfiasi.com (A.C.); cornelia.mircea@umfiasi.ro (C.M.); bianca.ivanescu@umfiasi.ro (B.I.); monica.hancianu@umfiasi.ro (M.H.); irina-macovei@umfiasi.ro (I.M.); 2 Centre of Advanced Research in Bionanoconjugates and Biopolymers Department, “Petru Poni” Institute of Macromolecular Chemistry, 41A Grigore Ghica Voda Alley, 700487 Iasi, Romania; moleavin.ioana@icmpp.ro; 3 Department of Pharmaceutical Botany, Faculty of Pharmacy, Iuliu Hațieganu University of Medicine and Pharmacy, 8 Victor Babeș Street, 400012 Cluj-Napoca, Romania; gheldiu.ana@umfcluj.ro

**Keywords:** *Aronia melanocarpa*, green synthesis, silver nanoparticles, antioxidant activity, photocatalytic activity, cytogenototoxicity

## Abstract

**Background/Objectives:** This study reports the green synthesis, optimization, characterization, and multifunctional evaluation of silver nanoparticles (AgNPs) using an ethanolic *Aronia melanocarpa* berry extract. The objective was to establish optimal synthesis conditions; assess the in vitro stability; and evaluate the antioxidant, photocatalytic, and photoprotective activities. **Methods**: The cytogenotoxic effects of the AgNPs were evaluated on *Triticum aestivum* roots. The AgNPs were synthesized via bioreduction using an ethanolic extract of *A. melanocarpa* under varied pH, AgNO_3_ concentration, extract/AgNO_3_ ratio, temperature, and stirring time, with optimization guided by UV–Vis spectral analysis. The AgNPs were further characterized by FTIR, DLS, TEM, and EDX. In vitro stability was evaluated over six months in different dispersion media (ultrapure water; 5% NaCl; and PBS at pH 6, 7, and 8). Biological assessments included antioxidant assays (lipoxygenase inhibition, DPPH radical scavenging, metal chelation, and hydroxyl radical scavenging), photocatalytic dye degradation, and SPF determination. **Results**: Optimal synthesis was achieved at pH 8, 3 mM AgNO_3_, extract/AgNO_3_ ratio of 1:9, 40 °C, and 240 min stirring. The AgNPs were spherical (TEM), well dispersed (PDI = 0.32), and highly stable (zeta potential = −40.71 mV). PBS pH 6 and 7 ensured the best long-term colloidal stability. The AgNPs displayed strong dose-dependent antioxidant activity, with superior lipoxygenase inhibition (EC_50_ = 18.29 µg/mL) and the effective photocatalytic degradation of dyes under sunlight. Photoprotective properties were confirmed through UV absorption analysis. The AgNPs showed a strong antimitotic effect on wheat root cells. **Conclusions**: The study demonstrates that *A. melanocarpa*-mediated AgNPs are stable, biologically active, and suitable for potential biomedical, cosmetic, and environmental applications, reinforcing the relevance of plant-based nanotechnology.

## 1. Introduction

Green synthesis techniques have emerged as a result of nanotechnology’s quest for environmentally friendly and sustainable processes, especially in the development of metal-based nanoparticles (NPs). Among these, silver nanoparticles (AgNPs) have garnered considerable interest given their distinct physicochemical characteristics and wide range of biological activities, such as photocatalytic, antioxidant, anticancer, antibacterial, antifungal, and antiviral effects [1,2,3]. Traditional AgNPs synthesis techniques frequently include processes that require energy and hazardous chemicals, which raise questions about their biocompatibility and potential effects on the environment [4]. Plant-mediated synthesis has, thus, evolved into a viable substitute, providing a simple, economical, and environmentally safe method of producing NPs [5].

*Aronia melanocarpa*, commonly known as black chokeberry, is a species of shrubs native to North America that is widely recognized for its exceptionally high content of bioactive compounds. Its berries are particularly rich in polyphenolic constituents, including phenolic acids, anthocyanidins, and procyanidins, which contribute to its potent antioxidant capacity [6]. Such natural compounds not only offer significant health-promoting effects but also make *A. melanocarpa* an ideal candidate for applications in green chemistry. In particular, the redox properties and caping abilities of its phytochemicals can be correlated with their potential to act as natural bioreducing and stabilizing agents in the synthesis of metal nanoparticles, including AgNPs. However, despite the species’ well-known phytochemical complexity, its inclusion in nanomaterial synthesis and functional evaluation still remains underexplored. Although preliminary studies have highlighted its bioactivity and suggested promising interactions at the nanoscale level, comprehensive investigations into its role as a biological agent in nanomaterial development, particularly regarding its stability, efficacy, and mechanisms of action, are still lacking in the current scientific literature [7,8].

In this study, we report the green synthesis of AgNPs using an ethanolic extract of *A. melanocarpa* berries, followed by comprehensive optimization and characterization of the obtained NPs. Furthermore, we investigate their colloidal stability in various media, antioxidant potential through multiple in vitro assays, photocatalytic dye degradation efficiency, photoprotective capacities, and cytogenotoxic effects. By establishing the optimal synthesis conditions and evaluating multifunctional properties, this work highlights the biomedical, cosmetic, and environmental relevance of *A. melanocarpa*-derived AgNPs, contributing to the growing field of plant-based nanosystems with promising applications.

## 2. Materials and Methods

### 2.1. Preparation and Characterization of Plant Extract

Extraction was employed following a method previously described. Briefly, 10 g of powdered *A. melanocarpa* berries (purchased from a local natural products store) were added to 100 mL 70% ethanol. The mixture was magnetically stirred for 30 min. at 40 °C followed by filtration, with the resulting plant extract being used afterward in AgNPs synthesis [7].

The ethanolic berry extract was first analyzed through the LC-MS method using an Agilent 1100 HPLC system equipped with a degasser, binary gradient pump, column thermostat, autosampler, and UV detector, and coupled with an Agilent 1100 mass spectrometer (Agilent, Santa Clara, CA, USA). Separation was performed in reverse phase using a Zorbax SB-C18 column with 3.5 μm particles while for identification the MS spectrophotometer with electrospray ion source was operated in the negative mode [9,10,11,12]. Two spectrophotometric methods were employed for the quantitative determination of total polyphenolic compounds and proanthocyanidins in the berry extract [13,14]. Furthermore, these quantifications were also assessed in the resulting supernatant after the synthesis of AgNPs and their separation by centrifugation.

### 2.2. Optimized Synthesis of AgNPs

To establish the optimal conditions in which the maximum yield of AgNPs is obtained, several parameters were varied and the synthesis was monitored by recording the UV-Vis spectra with a Specord 210 Plus spectrophotometer (Analytik Jena, Thuringia, Germany) in the wavelength range of 300–600 nm [15,16]. Therefore, several experiments were conducted by varying the pH (2, 6, and 8), AgNO_3_ concentration (1, 3, and 5 mM), ratio between plant extract and AgNO_3_ (9:1, 5:5, and 1:9), temperature (20, 40, and 60 °C), and time of stirring (5, 15, 30, 60, 90, 180, and 240 min). The pH was adjusted using HCl 0.1 M and NaOH 0.1 M. Further, the colloidal dispersion of AgNPs was obtained by implementing the selected optimal conditions. AgNPs dispersion was centrifugated afterward at 6800 rpm for 30 min. (Hettich Rotina 380 R centrifuge, Hettich, Tuttlingen, Germany) and washed two times with ultrapure water and centrifugated again, and the resulting sediment represented by AgNPs pellets was dried at 40 °C until constant mass and stored at 4 °C until further use.

### 2.3. In Vitro Stability of AgNPs

The evaluation of the in vitro stability of AgNPs followed a procedure previously described [7]. In summary, mixtures between AgNPs dispersions (5 mL) and different media (5 mL) were monitored through UV-Vis spectroscopy (300–600 nm UV-Vis spectra) at different time intervals (0, 7, 14, 21, and 28 days, and 6 months). The media used were represented by 5% NaCl solution and phosphate-buffered (PBS) saline solutions at pH 6, 7, and 8. Ultrapure water was included in the experiment as a control under identical circumstances. During the experiment, all the mixtures were stored at room temperature, in the dark.

### 2.4. Characterization of AgNPs

AgNPs characterization was performed through spectroscopic (UV-Vis, FTIR, DLS, EDX) and microscopic (TEM) techniques [7,17]. Confirmation of AgNPs synthesis was established by UV-Vis spectroscopy due to the surface plasmon resonance phenomenon—collective oscillations of metal nanoparticle electrons when exposed to light (SPR). The involvement of plant extract’s constituents in AgNPs synthesis as reducing and capping agents was analyzed by FTIR spectroscopy. The FTIR spectra of both *A. melanocarpa* berry extract and its derived AgNPs were recorded with a Bruker Vertex 70 spectrophotometer (Bruker, Billerica, MA, USA) in the spectral region between 4000 and 310 cm^−1^ with a resolution of 4 cm^−1^. The synthesized AgNPs colloidal dispersion was subjected to DLS analysis using the Delsa Nano Submicron Particle Size Analyzer (Beckman Coulter Inc., Fullerton, CA, USA). The analysis revealed the hydrodynamic diameter, polydispersity index, and zeta potential of the AgNPs in dispersion. AgNPs shapes and sizes were investigated through the TEM technique when images of the AgNPs were captured with a Hitachi High-Tech HT 7700 microscope (Hitachi High-Technologies Corporation, Tokyo, Japan). EDX analysis was employed to investigate the AgNPs elemental composition, for which the sample was deposited on an aluminum substrate and analyzed using a Quanta 200 environmental scanning electron microscope (SEM) with EDX (FEI Company, Brno, Czech Republic).

### 2.5. Phytotoxicity and Cytogenetic Analysis of AgNPs on Triticum aestivum

The phytotoxicity test was performed according to Vannini et al. with some modifications [18]. After a process of decontamination, the seeds of *Triticum aestivum* were left in water to inflate for 24 h and then the inflated seeds were placed in Petri dishes, on filter paper impregnated with AgNPs 10 mg/L, AgNPs 20 mg/L, aqueous *A. melanocarpa* berry extract 20 mg/L, ethanolic *A. melanocarpa* berry extract 20 mg/L, and water (control). The Petri dishes were kept at 24 °C and samples were added every day. On the 7th and 14th days, some plants were separated, and the shoots were measured.

Mitotic studies were conducted on root tips of germinated seeds post treatments. The samples were removed when radicles were 1.5–2 cm long. The root tips containing the meristem tissue were stained with acetocarmine [19] and then squashed. A number of approximately 2000 cells per sample was examined at 400× magnification using an Eclipse E400 microscope equipped with a Nikon D700 camera (Nikon, Tokyo, Japan). The mitotic index was calculated as the percentage of cells undergoing mitosis of the total number of cells scored. The number of cells in different mitotic stages (prophase, metaphase, anaphase, and telophase) was expressed as a percentage of the total dividing cells [20]. The visible aberrations in the chromosomes of the treated samples were also recorded.

### 2.6. Antioxidant Activity

The antioxidant potential of *A. melanocarpa* berry extract and their derived AgNPs was investigated through four in vitro spectrophotometric assays (lipoxygenase inhibition, DPPH radical scavenging, metal ion chelating, and hydroxyl radical scavenging activities). The methods employed followed procedures previously described in the literature [7,15]. In summary, lipoxygenase inhibition activity was evaluated by recording the decrease in absorbance values at 234 nm due to *A. melanocarpa* berry extract and its derived AgNPs capacities to block linoleic acid oxidation [21]. The berry extract and AgNPs’ scavenging activities of the unstable DPPH radical were spectrophotometrically assessed by examining the absorbance values at 517 nm [7]. To evaluate the investigated samples’ capacities to chelate metal ions (Fe^2+^), an experiment was conducted based on the formation of a pink complex between ferrozine and Fe^2+^ with an absorption maximum of 562 nm. Therefore, if chelating agents are present in the reaction media (*A. melanocarpa* berry extract and derived AgNPs) causes a decrease in absorption at 562 nm [22]. In the hydroxyl radical scavenging method, the protocol relies on the hydroxylation of salicylic acid by the hydroxyl radical released from the reaction between Fe^2+^ and H_2_O_2_ with the formation of a pink complex characterized by an absorption maximum at 562 nm [23]. The half-maximal effective concentration (EC_50_) was calculated for the samples which showed a percentage of activity over 50%. EC_50_ value was obtained through linear interpolation by taking into consideration the first values below and above 50%. Each experiment was performed in triplicate and the result was illustrated as average ± standard deviation.

### 2.7. Photocatalytic Activity

The green synthesized AgNPs’ potential to act as photocatalytic agents was explored towards two triphenylmethane dyes (malachite green and crystal violet) and a thiazine dye (methylene blue) [24]. In summary, the implemented protocol involved the sunlight irradiation of the AgNPs (4 mg) and 10 mg/L aqueous dye solution (20 mL) mixtures at room temperature (25 °C). The photocatalytic degradation of the dyes was monitored by recording the UV-Vis spectra in the wavelength range of 350–800 nm at several periods of time (0, 30, 60, and 90 min) of sunlight exposure under continuous magnetic stirring [25,26]. Samples of colloidal AgNPs dispersions in the dye solution were centrifuged (5000 rpm., 5 min) prior to each UV-Vis spectra recording, with the supernatant being subjected to the analysis. Concomitant, controls (malachite green, methylene blue, and crystal violet solutions without AgNPs) were included in the experiment under the same conditions [27]. The percentage of degradation of the organic dyes after 90 min. of exposure was calculated based on the Beer–Lambert law:

$$\%Degradation=[(C_{0}-C)/C_{0})]\times 100=[(A_{0}-C)/A_{0})]\times 100$$

where C_0_ and C are the dye concentrations in the initial moment and after 90 min. of exposure, respectively; A_0_ and A are the absorbance values for the dye—AgNPs mixtures recorded initially and after 90 min. of exposure.

### 2.8. Photoprotective Activity

The sunburn protection factor (SPF) analysis represents a spectrophotometric method relying on the determination of absorbance in the UV-B wavelength range (290–320 nm) every 5 nm [28,29]. For this test, two extract concentrations (0.01 g % and 0.02 g %) and three concentrations of AgNP colloidal solutions (0.005%, 0.01 g % and 0.02 g %) were considered. The SPF values for berry extract and derived AgNPs were calculated by using the Mansur equation:

$$SPF=CF\times \sum_{290}^{320}EE\left(\lambda \right)\times I\left(\lambda \right)\times Abs(\lambda )$$

where CF represents the correction factor (=10), EE is a constant that stands for the erythemogenic effect of radiation of wavelength λ, I is a constant that stands for the intensity of solar light of wavelength λ, and Abs is the absorbance value at wavelength λ.

## 3. Results

### 3.1. A. melanocarpa Berry Extract Characterization

Firstly, the ethanolic *A. melanocarpa* extract used in AgNPs synthesis and the supernatant resulting after AgNPs separation were subjected to phytochemical characterization in order to quantify the total phenolic and anthocyanin content. The total phenolic content was assessed by the Folin–Ciocalteu method, with the results being expressed as mg gallic acid equivalents (GAE)/mL sample. The quantification of anthocyanins was performed following a method described in the European Pharmacopoeia, with the results being expressed in cyanidine 3-glucoside mg %. The results showed that the ethanolic *A. melanocarpa* berry extract has a polyphenolic content of 3.669 mg GAE/mL, while the supernatant contained only 0.167 mg GAE/mL. Regarding anthocyanins content, a value of 87.14 mg cyanidine 3-glucoside % was registered for the ethanolic extract, while no anthocyanins could be detected in the supernatant.

Secondly, the LC-MS method was employed in order to identify the phenolic compounds involved in AgNPs synthesis. The following compounds were investigated: caffeic, caftaric, chlorogenic, 4-O-caffeoylquinic, p-coumaric, ferulic, gentisic, and sinapic acids; apigenin; fisetin; hyperoside; isoquercitrin; kaempferitrin; kaempferol; kaempferol-3-rhamnoside; luteolin; myricetin; patuletin; quercetin; quercitrin; rutoside; vitexin; and vitexin 2-O rhamnoside. Table 1 depicts the detected compounds.

### 3.2. Optimized Synthesis and Generation of AgNPs

**Figure 1:**
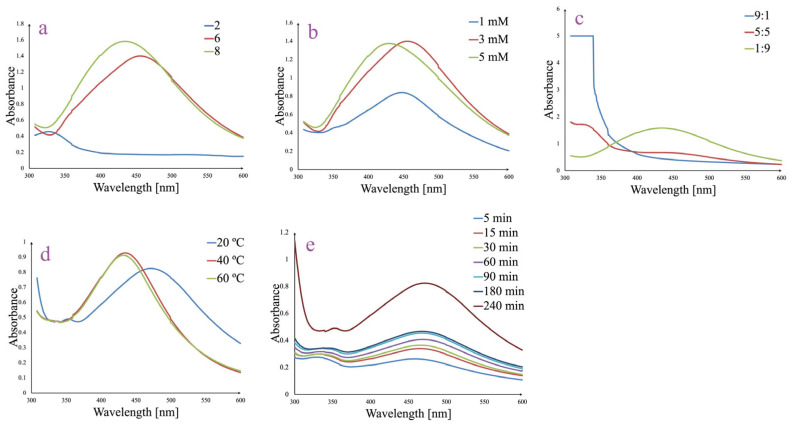
Effect of pH (**a**), AgNO_3_ concentration (**b**), ratio between plant extract and AgNO_3_ (**c**), temperature (**d**), and stirring time (**e**) over AgNPs synthesis.

The influence of the parameters known to affect the AgNPs green synthesis process was tested by employing several experiments which were monitored through UV-Vis spectroscopy in the wavelength domain between 300 and 600 nm. Thus, synthesis was assessed in various conditions of pH (Figure 1a), AgNO_3_ concentration (Figure 1b), ratio between plant extract and AgNO_3_ (Figure 1c), temperature (Figure 1d), and stirring time (Figure 1e). Each parameter was varied independently to identify optimal synthesis conditions that promote efficient AgNPs synthesis, indicated by a characteristic SPR peak in the 400–450 nm range. At the same time, visually, the change in the color of the solution from light yellow to dark brown was observed.

The pH of the reaction mixture significantly influenced the AgNPs formation (Figure 1a). At pH 2, the UV–Vis spectrum lacked a defined SPR peak, suggesting no AgNPs synthesis. In contrast, both pH 6 and pH 8 generated distinct SPR bands centered around 430–450 nm, which indicates successful AgNPs synthesis processes. The higher absorbance and sharper shape for the peak recorded for pH 8 suggest that alkaline conditions favor the reduction of Ag^+^ and stabilization of AgNPs.

Increasing the AgNO_3_ concentration enhanced the AgNPs formation, as evidenced by the rise in absorbance intensity of the SPR band (Figure 1b). The 1 mM AgNO_3_ sample showed a weaker SPR band, while 3 and 5 mM generated more intense and well-defined peaks. The highest value for absorbance was recorded for 3 mM AgNO_3_ (1.40 at λ_max_ = 450 nm), and therefore, this concentration was selected. These results indicate that a higher silver ion availability promotes a more efficient AgNPs synthesis. However, over a certain AgNO_3_ concentration, the synthesis process appears to be slightly impaired.

The volume ratio between plant extract and AgNO_3_ was also identified as a critical factor (Figure 1c). A 1:9 ratio of extract to silver nitrate yielded the most prominent SPR peak, indicating efficient AgNPs synthesis. At a 5:5 ratio, AgNPs synthesis was moderate, while the 9:1 ratio showed strong absorbance below 350 nm, suggesting the presence of unreacted phytocompounds from the ethanolic berry extract and no AgNPs synthesis. These results emphasize the need for sufficient silver ions to support the complete reduction and formation of AgNPs.

Temperature variations affected the intensity and shape of the SPR band. While all the temperatures (from 20 to 60 °C) led to SPR peak formation, the spectra at 40 and 60 °C were sharper and slightly red-shifted compared to 20 °C, suggesting that higher temperatures enhance reaction kinetics and lead to more uniform nanoparticle formation. Considering the green synthesis principles and the fact that between the absorbances at λ_max_ = 437 nm a minor difference was absorbed (0.92 vs. 0.91 for 40 and 60 °C, respectively), the temperature of 40 °C was selected as optimal.

Reaction time also had a significant impact on AgNPs synthesis. Within the first time frame (5–30 min), UV-Vis spectra showed only low absorbance and broad peaks. A well-defined SPR peak appeared after 60 min (0.4 absorbance at λ_max_ = 470 nm) and increased gradually up to 240 min (0.82 absorbance at λ_max_ = 470 nm), indicating that extended reaction times promote the more complete reduction of Ag^+^ and improved stability. Notably, the SPR peak became sharper and more intense with increasing time, confirming the progressive formation of well-dispersed AgNPs.

Therefore, the most effective synthesis was achieved under the following optimized conditions: pH 8, AgNO_3_ concentration of 3 mM, ratio between plant extract and AgNO_3_ of 1:9, temperature of 40 °C, and stirring time of 240 min.

### 3.3. AgNPs In Vitro Stability

**Figure 2:**
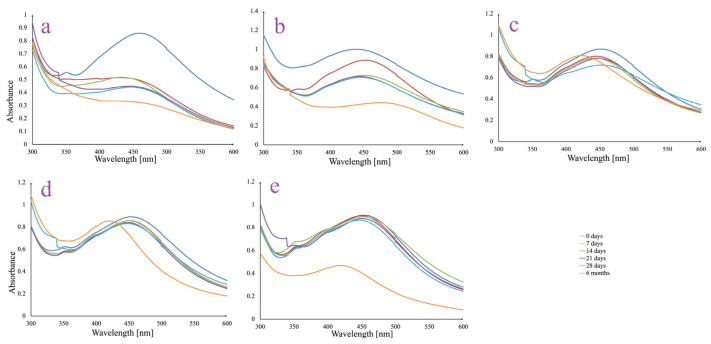
UV-Vis spectra of AgNPs colloidal dispersions in different media: ultrapure water (**a**); 5% NaCl solution (**b**); phosphate-buffered saline (PBS) solution, pH 6 (**c**); phosphate-buffered saline (PBS) solution, pH 7 (**d**); and phosphate-buffered saline (PBS) solution, pH 8 (**e**).

The long-term colloidal stability of the AgNPs synthesized using ethanolic *A. melanocarpa* berry extract was assessed in different dispersion media over a 6-month period (Figure 2).

The AgNPs dispersed in ultrapure water (control sample) showed a progressive decline in SPR peak intensity (~440–450 nm) over time, along with a broadening trend (Figure 2a). By 6 months, the peak had significantly decreased, suggesting moderate instability and gradual aggregation. The reduction in intensity indicates a decline in colloidal stability in the absence of ionic or buffering components.

The AgNPs were least stable in a 5% NaCl solution (Figure 2b). A substantial decrease in SPR peak intensity was observed as early as day 7, with further flattening and broadening by 6 months. This behavior is attributed to high ionic strength causing particle agglomeration and destabilization, leading to significant sedimentation or aggregation.

In PBS at pH 6 (Figure 2c), the SPR peak remained relatively stable during the first 28 days. However, a slight red-shift and broadening were detected over time. By 6 months, the peak maintained its intensity along with a displacement phenomenon of λ_max_ towards a shorter wavelength, suggesting a small degree of aggregation, likely influenced by the acidic pH and ionic environment.

PBS at neutral pH supported good AgNPs stability (Figure 2d). The SPR peak remained well defined with minimal shifts over the 28-day period. Similarly to PBS pH 6, at 6 months, a blue shift may be observed, but the peak keeps its shape and height, indicating relatively stable AgNPs in dispersion and minimal aggregation under physiologically relevant conditions.

PBS at pH 8 provided good stability only for the first 28 days (Figure 2e). Even though the SPR peak remained sharp in the first 28 days, at 6 months a significant decrease in intensity may be noted along with a blue shift in the peak. This suggests that mild alkaline pH, combined with buffering capacity, may preserve the AgNPs dispersion only for a limited period of time.

### 3.4. AgNPs Characterization

#### 3.4.1. FTIR Analysis

**Figure 3:**
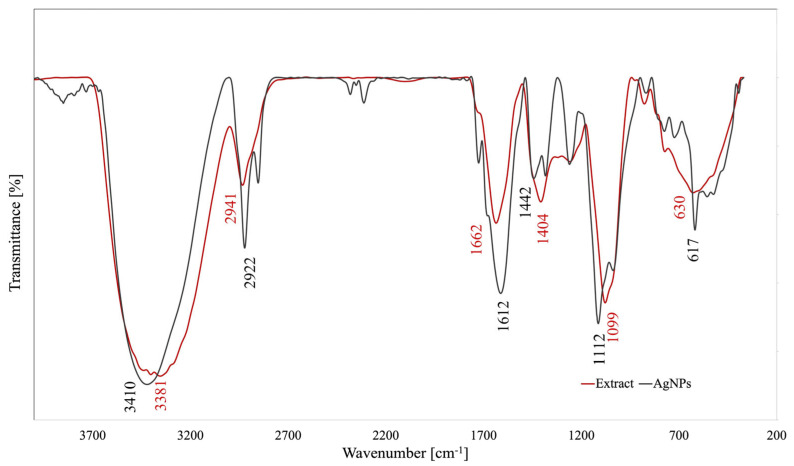
FTIR spectra of *A. melanocarpa* berry extract and its derived AgNPs.

FTIR analysis is an important tool used in the elucidation of the phytocompounds (by their functional groups) involved in the AgNPs biosynthesis as reducing and/or capping agents. Both the FTIR spectra of *A. melanocarpa* berry extract and of its derived AgNPs are illustrated in Figure 3.

The FTIR spectra of the ethanolic berry extract show an important absorption band at wavenumber 3381 cm^−1^ corresponding to the stretching vibration of -OH groups from alcohols, phenols, and polysaccharides [7,30]. The peak at 2900 cm^−1^ is attributed to C-H stretching vibration, while the band at 1662 cm^−1^ could be associated with N-H stretching vibration in amines overlapped with -C=C- vibrations in aromatic rings [15]. The =C-O-C stretching vibrations of flavonoids may be identified through the band observed at 1404 cm^−1^ [30]. Further, a broad band appeared at 1099 cm^−1^ (C-O stretching vibrations), suggesting the presence of various alcohols, esters, ethers, and carboxylic acids from terpenoids and flavonoids [7]. The presence of aromatic C-H out-of-plane bending vibrations is indicated by peaks located in the region between 900 and 600 cm^−1^ [20]. The AgNPs spectra show the same peaks with shift effects and intensity discrepancies (3410, 2922, 1612, 1442, 1112, and 617 cm^−1^), suggesting the involvement of the extracts’ compounds in AgNPs synthesis.

#### 3.4.2. DLS Analysis

**Figure 4:**
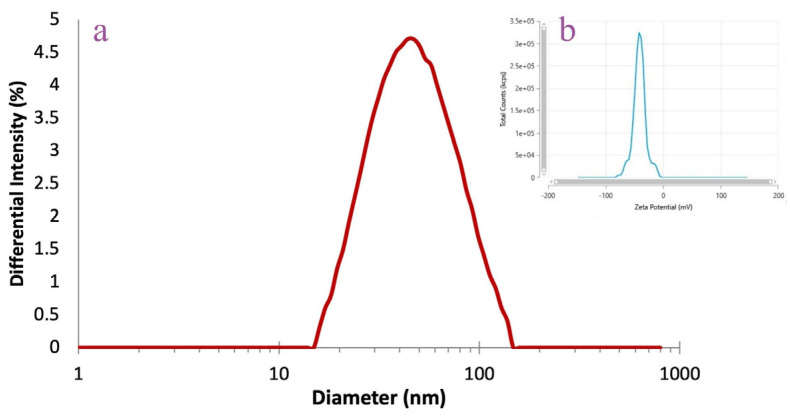
DLS analysis of AgNPs: hydrodynamic diameter (**a**) and zeta potential (**b**).

The DLS analysis was performed to assess the average AgNPs hydrodynamic diameter, polydispersity index, and zeta potential (Figure 4). The *A. melanocarpa* berry extract-derived AgNPs were characterized by values of 43.68 nm for hydrodynamic diameter, 0.32 for polydispersity index, and −40.71 mV for zeta potential.

#### 3.4.3. TEM Analysis

**Figure 5:**
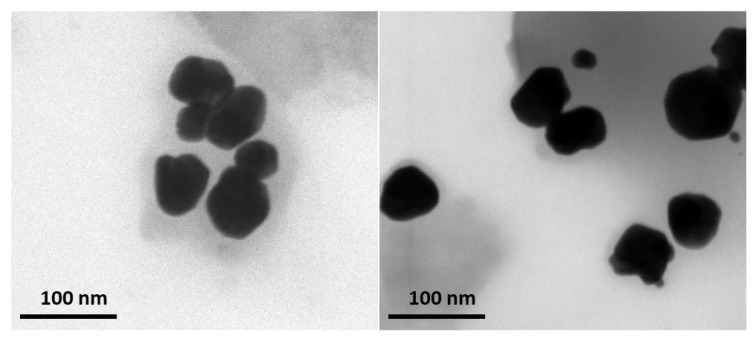
TEM images of AgNPs acquired at 200,000× magnification.

The morphological characteristics of the AgNPs were assessed by the TEM technique. TEM micrographs of the *A. melanocarpa* berry extract-derived AgNPs (Figure 5) show that the green synthesized particles are well dispersed with a spherical shape with homogeneous sizes and shapes and with a light gray colored area corresponding to AgNPs margins.

#### 3.4.4. EDX Analysis

**Figure 6:**
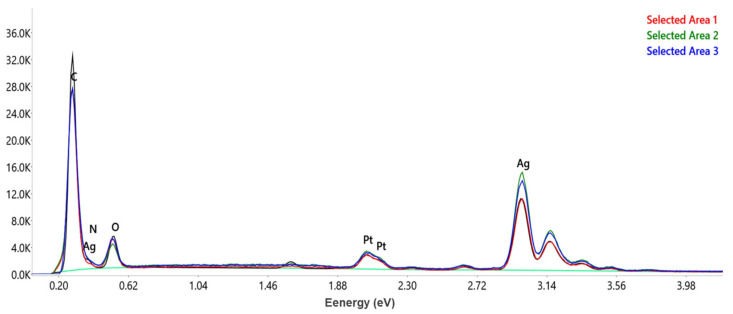
EDX spectra of AgNPs recorded in three different areas.

The EDX analysis was performed in order to achieve information regarding the qualitative and quantitative chemical composition of the *A. melanocarpa* berry extract-derived AgNPs surface. The EDX spectra (Figure 6) of the AgNPs revealed a strong signal for metallic silver (Ag) at 3 keV, which confirmed the formation of AgNPs (bioreduction of Ag^+1^ to Ag^0^). Peaks associated with C, N, and O were also present on the EDX spectra, confirming the presence of plant extract compounds that are attached to the AgNPs surface performing as capping agents.

The quantitative analysis of three different areas of the AgNPs surface shows that Ag is the major element with an average of 74.06 wt %, followed by C (14 wt %), O (4 wt %), and N (0.26 wt %). The element Pt is also present most probably due to the sample’s contamination during the preparation process [15].

### 3.5. AgNPs Phytotoxicity and Cytogenetic Analysis

**Figure 7:**
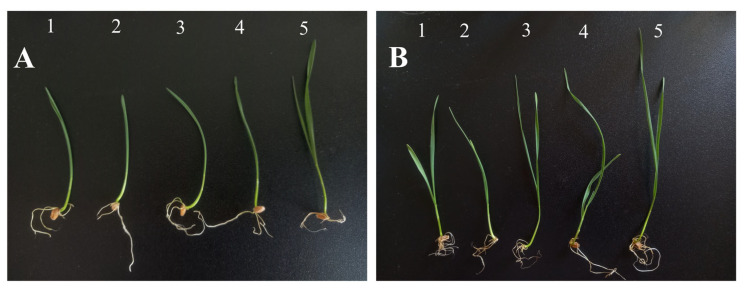
Effect of AgNPs 10 mg/L (1), AgNPs 20 mg/L (2), aqueous *A. melanocarpa* berry extract 20 mg/L (3), ethanolic *A. melanocarpa* berry extract 20 mg/L (4), and water/control (5) on wheat growth after 7 (**A**) and 14 days (**B**).

The preliminary phytotoxicity study on *Triticum aestivum* was determined by measuring the growth of the shoots after 7 and 14 days (Figure 7).

The results show that the dimensions of shoots depend on the medium used and decrease in the following order: water/control > ethanolic *A. melanocarpa* berry extract 20 mg/L > aqueous *A. melanocarpa* berry extract 20 mg/L > AgNPs 10 mg/L > AgNPs 20 mg/L. As compared to the extracts, the AgNPs suppressed wheat growth in a dose-dependent manner and the roots showed a tendency to reject the germination substrate (especially after 14 days).

**Figure 8:**
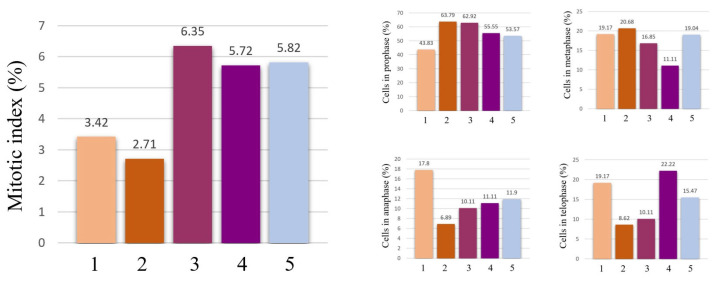
Mitotic index values and percentages of cells in mitosis stages in wheat root meristems exposed to AgNPs 10 mg/L (1), AgNPs 20 mg/L (2), aqueous *A. melanocarpa* berry extract 20 mg/L (3), ethanolic *A. melanocarpa* berry extract 20 mg/L (4), and water/control (5).

**Figure 9:**
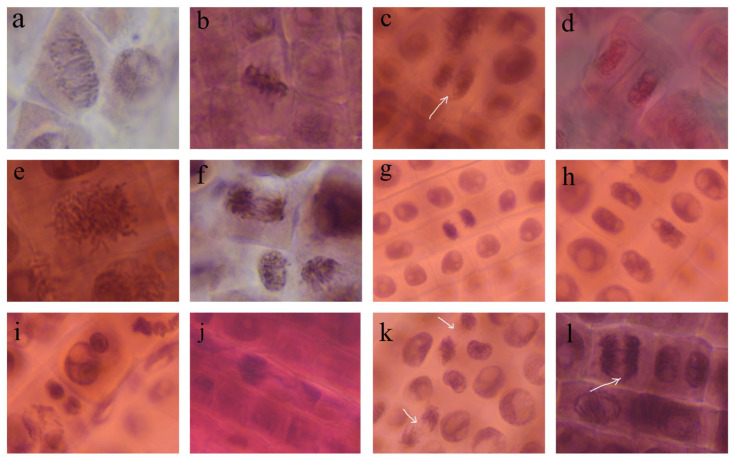
Normal stages of mitosis (**a**–**d**) and chromosomal aberrations (**e**–**l**) in *Triticum aestivum* root meristems, at 400× magnification: (**a**–**d**) normal prophase, metaphase, anaphase, and telophase in control; (**e**) spindle disturbance in metaphase (AgNPs 20 mg/L); (**f**) anaphase with multiple bridges (AgNPs 10 mg/L); (**g**) sticky telophase with one bridge (AgNPs 10 mg/L); (**h**) binucleate cell (AgNPs 10 mg/L); (**i**) micronuclei (AgNPs 10 mg/L); (**j**) sticky metaphase with lost chromosome (AgNPs 20 mg/L); (**k**) spindle disturbance and laggard chromosome at anaphase (AgNPs 10 mg/L); (**l**) chromosome bridge at anaphase (AgNPs 20 mg/L).

The effects of the AgNPs and the *A. melanocarpa* extracts on cell division and chromosome behavior are summed up in Figure 8 and Figure 9. The AgNPs significantly decreased the number of dividing cells, and therefore, the mitotic index. The effect is more pronounced for the AgNPs 20 mg/L concentration, where the mitotic index is reduced to less than half that of the control, suggesting that the toxicity is dependent on the concentration of AgNPs. On the other hand, the *A. melanocarpa* extracts do not hinder cell division, but actually stimulate it, as is the case of the aqueous extract. Regarding the stages of cell division, AgNPs 20 mg/L significantly reduces the number of cells in anaphase and telophase, while increasing the number of cells in prophase. The AgNPs decrease the number of cells in prophase.

Chromosomal aberrations were observed in wheat radicle cells after treatment with AgNPs, such as un-oriented chromosomes in disturbed metaphase, chromosomal stickiness, chromatin bridges, lost chromosomes, laggard chromosomes at anaphase, and micronuclei at interphase and binucleate cells.

### 3.6. AgNPs Antioxidant Activity

**Figure 10:**
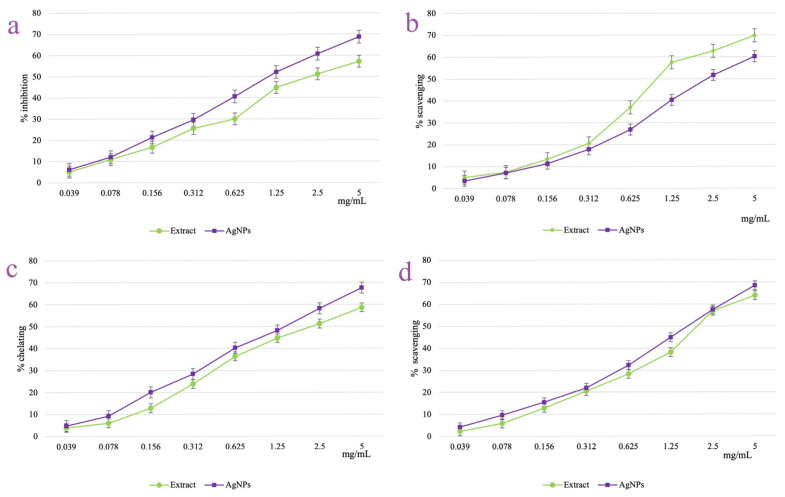
Antioxidant activity of *A. melanocarpa* berry extract and its derived AgNPs: lipoxygenase inhibition assay (**a**), DPPH radical scavenging test (**b**), metal ion chelating activity (**c**), and hydroxyl radical scavenging test (**d**).

The antioxidant potential of *A. melanocarpa* berry extract and its derived AgNPs were explored through four different in vitro assays which quantified the lipoxygenase inhibition percent (Figure 10a), the DPPH radical scavenging activity (Figure 10b), the metal (Fe^2+^) ion chelating efficiency (Figure 10c), and the hydroxyl radical scavenging capacity (Figure 10d). Both the berry extract and derived AgNPs showed a dose-dependent increased activity in all four experiments employed. The inhibition of lipoxygenase activity of the AgNPs increased from 6.13 ± 0.08 (at 0.039 mg/mL) to 68.74 ± 1.06% (at 5 mg/mL). In a similar manner, the AgNPs scavenging activities of DPPH and hydroxyl radicals and metal ion chelation capacity were improved from 3.57 ± 0.11, 4.24 ± 0.07, and 4.80 ± 0.09%, respectively (at 0.039 mg/mL), to 60.26 ± 0.26, 68.46 ± 0.08, and 68.38 ± 0.02%, respectively (at 5 mg/mL). For all the tested concentrations, the AgNPs activity was superior to the one shown by the berry extract, with the exception of the DPPH radical scavenging test. Even so, at the highest tested concentration (5 mg/mL), both berry extract and AgNPs scavenged the DPPH radical with an appropriate efficiency of 69.87 ± 0.54 and 60.26 ± 0.26 mg/mL, respectively.

As all the samples showed activities higher than 50% on the tested concentration range, the EC_50_ values were calculated in all the cases (Table 2). According to the EC_50_ values, the most intense activity was recorded for the AgNPs in the lipoxygenase inhibition assay (EC_50_ = 18.29 ± 0.52 μg/mL).

### 3.7. AgNPs Photocatalytic Activity

**Figure 11:**
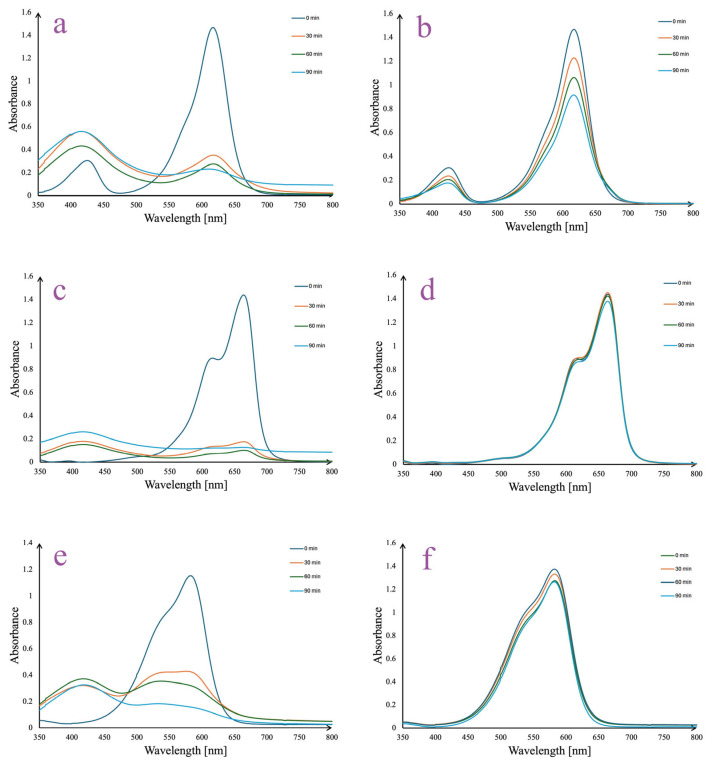
*A. melanocarpa* berry extract-derived AgNPs photocatalytic reduction in malachite green (**a**), methylene blue (**c**), and crystal violet (**e**) in time vs. controls: malachite green (**b**), methylene blue (**d**), and crystal violet (**f**) solutions.

The degradation of malachite green, methylene blue, and crystal violet by *A. melanocarpa* berry extract-derived AgNPs under sunlight irradiation is depicted in Figure 11. The process of dye degradation was monitored by recording the decrease in absorbances at the wavelengths of the most important peaks (λ_max_) on the analyzed dye spectra. For all the samples, it may be observed that AgNPs have a rapid photocatalytic effect under sunlight exposure, as the most important decrease in absorbances takes place in the first 30 min. Thus, after 30 min of stirring in sunlight, the absorbances decreased from 1.44 to 0.35 at λ_max_ = 619 nm (Figure 11a), from 1.43 to 0.15 at λ_max_ = 666 (Figure 11c), and from 1.52 to 0.42 at λ_max_ = 582 nm (Figure 11e) for malachite green, methylene blue, and crystal violet solutions, respectively. Minor changes in the absorbance values appeared in time (90 min), suggesting that a maximal effect has been already reached after 30 min of reaction. At the same time, for the control samples insignificant changes appeared for methylene blue (Figure 11d) and crystal violet (Figure 11e) solutions, while malachite green showed a minor decrease in absorbance from 1.45 (0 min) to 1.21 (30 min) at λ_max_ = 619 nm (Figure 11b).

### 3.8. AgNPs Photoprotective Activity

The sunscreen protection potential of the AgNPs was assessed by calculating the SPF values of both the *A. melanocarpa* berry extract and its synthesized AgNPs using a spectrophotometric method (Table 3).

It was observed that the SPF values increased proportionally with the concentration of the samples. At a concentration of 0.01%, the SPF value for the AgNPs was double compared to the extract, while at 0.02%, the AgNPs were three times more active than the extract. The AgNPs at 0.005% concentration offered sun protection almost equal to that expressed by the extract at a concentration of 0.02%.

## 4. Discussion

The systematic optimization of AgNPs synthesis using *A. melanocarpa* berry extract revealed that reaction conditions significantly influence nanoparticle formation, as reflected by the intensity and position of the SPR bands. Conditions such as pH 8, 3 mM AgNO_3_ concentration, 1:9 volume ratio of plant extract/AgNO_3_, 40 °C temperature, and stirring time of 240 min led to UV–Vis spectra which exhibited a high, sharp, and well-defined SPR peak centered around 440–450 nm, characteristic of uniformly distributed AgNPs. These results indicate that alkaline pH, sufficient silver ion concentration, a high silver/extract ratio, elevated temperature, and prolonged reaction time favor the efficient reduction of Ag^+^ ions and stabilization of AgNPs. The same conditions were observed for AgNPs synthesis from aqueous extract, except for the time needed to complete the synthesis reaction [7]. The different time required for a complete synthesis, which was higher for the ethanolic extract (240 min.), could be explained by the higher quantity of the extracted compounds readily available for AgNPs synthesis in an ethanolic vs. an aqueous extract [31,32].

According to literature data, compounds such as flavonoids, phenolics, terpenoids, alkaloids, tannins, saponins, carotenoids, coumarins, quinones, and lignans participate in the processes of the reduction and stabilization of NPs [33]. Also, the chemical composition of plants depends on climatic and environmental conditions, while the chemical composition of extracts also depends on extraction conditions [34,35]. *A. melanocarpa* contains polyphenolic compounds (flavonoids, phenolic acids, and tannins), carbohydrates, organic acids, proteins, amino acids, carotenoids, fatty acids, vitamins, and minerals [6,34]. Given the complex composition of *A. melanocarpa,* it is difficult to precisely establish which compounds participate in the AgNPs synthesis. In our research, we observed that the total phenolic content in the ethanolic extract was higher compared to the aqueous extract and in both cases, the content decreased in the supernatant. The LC-MS analysis confirmed these observations for compounds such as gentisic acid, hyperoside, isoquercitrin, rutoside, quercetin, and quercitrin. For chlorogenic and 4-O-caffeoylquinic acids, the quantities in the aqueous extract were higher than in the ethanolic extract; likewise, in the supernatant, the quantities were smaller for the ethanolic extract [7]. Moreover, anthocyanins were evaluated in the ethanolic extract but they could not be detected in the supernatant. Consequently, polyphenols and anthocyanins represent classes of compounds that seem to be responsible for the synthesis and capping of AgNPs obtained from *A. melanocarpa* berry extract. This affirmation is confirmed also by the FTIR, DLS, and TEM analysis. Generally, the FTIR spectra of the AgNPs showed similar peaks to the extract, as well as shifts, new peaks, or variations in the transmittance values. This observation suggests that phytocompounds from the ethanolic *A. melanocarpa* berry extract are involved in AgNPs synthesis as both the reducing and capping agents [15,17,20]. Moreover, the light gray-colored area corresponding to AgNPs margins observed by the TEM analysis may be attributed to the compounds in *A. melanocarpa* berry extract adhered to the AgNPs surface (capping agents) [30]. Furthermore, during the EDX analysis, the presence of the Ag characteristic peak confirmed the synthesis of the AgNPs, along with other identified elements, such as C, N, and O that belong to the phytocompounds found on the surface of the NPs that fulfill their role as capping agents [36].

Capping agents play an important role in the stability of NPs, a fact confirmed by DLS analysis, along with the estimated size of AgNPs in colloidal suspension and their dispersion, respectively. The colloidal AgNPs dispersion showed a narrow range of particle sizes and high uniformity, as these characteristics are indicated when the PDI value is low (below 0.7) [37]. The zeta potential shows the electric charge of the AgNPs surface and therefore predicts the stability of an AgNPs dispersion. According to guidelines, the *A. melanocarpa* berry extract-derived AgNPs (zeta potential = −40.71 mV) fall in the category of highly stable dispersions (zeta potential > ±30 mV) mV. The stability of the AgNPs in dispersion is supported by the negative–negative repulsive force between them [38,39].

Moreover, we evaluated the stability of the AgNPs in different media by monitoring changes in the UV–Vis absorption spectra, focusing on the SPR peak characteristics: intensity, position, and shape, which may indicate AgNPs integrity and aggregation behavior. The in vitro stability study of the AgNPs revealed that the dispersion medium plays a critical role in maintaining nanoparticle integrity over time. Among the tested environments, PBS demonstrated the highest stability after 28 days, but with changes in the SPR band after 6 months. For example, in the case of PBS at pH 6, minor signs of aggregation appear over time, and in the case of PBS at pH 8, both major signs of aggregation and an important decrease in absorbance value can be observed. Instead, for PBS at neutral pH 7, even after 6 months, good stability may be observed, indicating that physiological conditions are favorable for preserving the AgNPs dispersion. It is well established that both acidic and basic conditions can induce the aggregation of AgNPs, with the underlying mechanisms varying depending on the pH. AgNPs stabilized by plant-derived biomolecules tend to lose stability in acidic media due to changes in surface chemistry. Biomolecules such as carboxylic acids, polyphenols, and proteins present in plant extracts act both as reducing agents and surface-capping ligands. Under acidic conditions, the protonation of their functional groups weakens their interaction with the nanoparticle surface, diminishing stabilization and facilitating aggregation or degradation [40,41]. Moreover, acidic conditions suppress the dissociation of weak acidic groups in the biomolecular shell, enhancing hydrogen bonding and promoting AgNPs aggregation. At high pH, AgNPs stability initially improves due to the efficient dissociation of acidic functional groups in plant-derived biomolecules [42]. This effect was observed in our study during the first 28 days. However, extended exposure to basic conditions can result in nanoparticle degradation, driven by secondary silver reactions and the breakdown of the biological capping agents [43,44]. On the contrary, significant instability and aggregation were observed in high-ionic-strength solutions, particularly in 5% NaCl, where the SPR peak diminished substantially. Ultrapure water showed moderate stability but lacked the buffering capacity needed for long-term colloidal maintenance. Overall, these results highlight that neutral buffered environments are optimal for preserving the physicochemical stability of AgNPs, making such conditions suitable for further biomedical or environmental applications.

The mechanism by which AgNPs induce genotoxicity is multi-modal. AgNPs uptake induces oxidative stress in plants by generating reactive oxygen species (ROS). Excess ROS leads to DNA damage causing mutations or strand breaks, protein and lipids damage, alteration of membrane permeability, deactivation of enzymes, and, ultimately, apoptosis (programmed cell death) [45,46]. AgNPs and released Ag^+^ ions may bind directly to DNA, interfering with replication and transcription. Silver ions can replace essential metal ions in enzymes, inhibiting DNA repair enzymes or polymerases [47,48]. AgNPs can interfere with the mitotic spindle apparatus, leading to the mis-segregation of chromosomes. The inhibition or delay of mitotic progression can result in aneuploidy or polyploidy [49].

The accumulation of cells in prophase indicates that treatment with AgNPs 20 mg/L disrupts early mitotic events, delaying or blocking the transition to metaphase. This may be due to DNA damage, incomplete mitotic signaling, spindle checkpoint activation, inhibition of spindle formation, and chromosome condensation [48,50]. Spindle assembly checkpoint activation due to improper chromosome attachment to spindle fibers prevents the transition into anaphase, which may explain the small numbers of cells in anaphase-telophase in wheat grains treated with AgNPs 20 mg/L. Treatment with AgNPs 10 mg/L reduced prophase cell counts, suggesting that it prevented cells from reaching or initiating prophase. This is performed primarily by arresting the cell cycle before mitosis in the G1 or G2 phase due to DNA damage and the activation of cell cycle checkpoints or by inducing programmed cell death if the damage is too severe [48].

Other studies indicate that treatment with AgNPs inhibits mitosis by interfering with early mitotic events, particularly during prophase, by disrupting chromatin structure and spindle formation [20,51]. A significant reduction in the mitotic index in root tips cells of *Allium cepa*, *Vicia faba*, and *Triticum aestivum* was reported by different authors following the exposure to AgNPs [19,20,52,53]. The extent of these effects depends on factors such as nanoparticle concentration, size, surface charge, exposure duration, and the specific plant species involved [53].

AgNPs synthesized from *A. melanocarpa* also induced chromosomal aberrations in the wheat meristem cells: disturbed metaphase with clumped and un-oriented chromosomes, sticky anaphase and telophase, chromatin bridges at anaphase and telophase, laggard chromosomes in anaphase, micronuclei in interphase, and binucleate cells. Chromosome stickiness refers to abnormally clumped or glued-together chromosomes, losing their individual identities and failing to segregate properly during mitosis. This is usually a cellular dysfunction of irreversible type, leading to mitotic arrest, chromosomal breakage, or cellular death [54].

Similarly to our findings, *Corallina elongata*-derived AgNPs caused different abnormalities, such as chromatin bridges, uncoiling chromosomes, stickiness, multiple nuclei, micronuclei, elongation, cell wall damage, and ghost cells [19]. These AgNPs also reduced the mitotic index and increased the number of aberrations in wheat meristem cells in a manner directly proportional to the concentration of AgNPs and the contact time. In another study, *Picea abies* and *Pinus nigra* bark extract-derived AgNPs depressed mitosis in onion root cells and produced vagrant chromosomes and multiple bridges in anaphase [20]. Debnath et al. reported that gold and silver NPs significantly decreased the mitotic index in onion root meristem and induced stickiness of chromosomes, chromosome breaks, nuclear notch, and clumped chromosomes [51]. Therefore, the synthesized AgNPs manifest genotoxic effects on *Triticum aestivum* root cells as supported by the significant inhibition of mitosis depending on concentration and the presence of chromosomal aberrations.

Apart from having an impact on the production of AgNPs or the stabilization of the resulting NPs, the compounds identified in the extract (both qualitatively and quantitatively) additionally contribute to the biological activities.

The antioxidant activities of *A. melanocarpa* extracts are dependent on the content of polyphenols and cyanosides [55,56]. Due to their rich content in polyphenolic compounds and anthocyanins, *A. melanocarpa* berries and extracts have a higher antioxidant capacity than many fruits from the human diet or are used in the pharmaceutical industry for preparations with antioxidant activity [57].

Moreover, the literature explains the discrepancies in antioxidant activities especially by the use of various solvents, extraction methods, and conditions, or the different compositions of extracts (for example the quantity of polyphenols is greater in the ethanolic extract compared with the aqueous one, the predominant form of polyphenols—aglycones in the ethanolic extract and glycosylated forms in the aqueous extract, respectively) [58,59]. Thus, when comparing the results obtained for the AgNPs synthesized with the ethanolic extract with our previous work [7] when AgNPs were obtained with an aqueous *A. melanocarpa* berry extract, some differences can be observed in terms of antioxidant activity. In ion chelating efficiency and lipoxygenase inhibition assays, for both the aqueous extract and derived AgNPs, the EC_50_ values could not be calculated. On the other hand, for the ethanolic extract and derived AgNPs the EC_50_ could be calculated, with the lowest value being observed for AgNPs. There are studies in the literature in which extracts of *A. melanocarpa* have been used to block other enzymes involved in the oxidative stress process, such as xanthine oxidase or nitroxide synthase [60]. The inhibition capacity of the enzyme is higher when using alcoholic extracts compared to aqueous ones, which explains the values below 50% of the inhibition capacity determined for the aqueous extract and its corresponding AgNPs [61]. For hydroxyl radical scavenging capacity, the EC_50_ value was recorded for both types of AgNPs, while for extracts, only for the ethanolic one, which can be explained by the higher degree of solubility of polyphenolic compounds in alcoholic and hydroalcoholic solutions [56,62]. Regardless of the employed method, we see a dose-dependent antioxidant activity for every sample. Additionally, for the DPPH radical scavenging, the extract’s EC_50_ values were higher than those of their generated AgNPs activity for both types of extracts.

The best antioxidant activity expressed by EC_50_ was registered for lipoxygenase inhibition, followed by hydroxyl radical scavenging capacity and ion chelating efficiency. In silico studies have shown that the enzyme inhibition process can also be induced by compounds with hydrophobic groups that can influence the spatial structure of the enzyme. The inhibition phenomenon is also dependent on the position of the substituents on the aromatic nucleus [63]. Compounds that present substituents in the para position of benzene nuclei in the structure of polyphenols have a higher capacity to inhibit lipoxygenase [64]. The lipid peroxidation inhibition activity of the *A. melanocarpa* extracts is also evidenced by in vivo studies in which it was observed that the alcoholic extract has the ability to reduce the plasma level of malondialdehyde (a marker of lipid peroxidation) [62] or oxidative stress resulting from lipid overload of the liver [65]. Furthermore, the metallic core of AgNPs concentrates polyphenols and anthocyanins through their stabilizing interactions during nanoparticle formation. Thus, the increased metallic surface area of nanoparticles enhances antioxidant loading, improving their overall antioxidant activity. Moreover, several studies have demonstrated that silver metallic core can further improve the antioxidant activity of plant extracts through catalytic mechanisms, leading to an overall antioxidant capacity that exceeds that of the extract alone [66,67]. On the other hand, the transformation of bioactive compounds during the synthesis of nanoparticles further enhances their antioxidant activities [68,69].

This synergistic interaction underscores the crucial role of both the metallic core and the phytochemical capping agents in contributing to the enhanced antioxidant potential of the resulting nanomaterials.

Considering the antioxidant activity of the synthesized AgNPs, and the well-known fact that antioxidants from plants have the ability to protect skin tissues against sun damage [70], we also analyzed the possible photoprotective activity. According to the FDA, an SPF between 2 and 12 offers minimal protection [71] and according to European legislation, the minimum SPF value should be 6 [72]. Ghazwani et al. prepared a gel that contained methyl-anthranilate-loaded AgNPs and determined the comparative SPF value of AgNPs, methyl-anthranilate gel, and methyl-anthranilate-loaded AgNPs gel. The SPF values of 9.81, 24.9, and 35.75 suggested an increase in sun protection after the incorporation of methyl-anthranilate loaded into AgNPs. Therefore, our *A. melanocarpa* berry extract-derived AgNPs 0.02% could be a promising candidate as a sunscreen ingredient, considering its incorporation into a cosmetic product or combination with other compounds for a synergistic effect, and along with pre-clinical, clinical, dermatological tests [29]. The AgNPs demonstrate photoprotective activity due to their distinctive optical properties, particularly their capacity to absorb and scatter ultraviolet (UV) radiation. A key contributor to this effect is the surface SPR phenomenon exhibited by the AgNPs [73]. The size and morphology of the nanoparticles are also critical factors, as smaller particles with higher surface area/volume ratios enhance UV light interaction. Furthermore, the stabilization of the AgNPs by the plant extracts significantly enhances their photoprotective potential. Phytochemicals such as flavonoids, tannins, and other phenolic compounds not only mediate the reduction of silver ions during nanoparticle synthesis but also improve UV absorption and contribute to reactive oxygen species (ROS) scavenging under light exposure [74,75,76].

The mechanism underlying the observed photocatalytic activity can be attributed to the generation of electron-hole pairs on the AgNPs surface upon light irradiation. When AgNPs are exposed to light, they absorb photons, leading to the excitation of electrons and the formation of electron-hole (e^−^/h^+^) pairs. The excited electrons (e^−^) and holes (h^+^) participate in redox reactions that generate ROS such as hydroxyl radicals (•OH) and superoxide anions (O₂^−^•). These ROS are capable of attacking dye molecules, breaking their chromophoric structures, and leading to mineralization into less harmful products. Additionally, the SPR exhibited by the AgNPs enhances their light absorption and promotes efficient charge separation, thereby increasing ROS production and improving overall photocatalytic performance [77,78,79].

When compared to similar studies, the AgNPs synthesized in this work exhibit comparable or superior photocatalytic performance. For instance, AgNPs synthesized using a stem *Nepeta leucophylla* methanolic extract achieved 82.8% degradation of methylene blue under visible light [80], while other research utilizing black tea extract for AgNPs synthesis reported efficient degradation of malachite green under solar irradiation [81]. In another study, *Ruellia tuberosa* leaf extract—derived AgNPs achieved 87% degradation of crystal violet [82]. The results obtained in the present study are in line with these findings, supporting the efficiency and versatility of photosynthesized AgNPs for environmental remediation applications.

## 5. Conclusions

This study successfully demonstrates the green synthesis of AgNPs using an ethanolic *Aronia melanocarpa* berry extract as a bioreducing and stabilizing agent. The synthesis process was optimized, with the most efficient nanoparticle formation observed at pH 8, 3 mM AgNO_3_, a plant extract/metal precursor ratio of 1:9, 40 °C, and 240 min of reaction time. The resulting AgNPs were confirmed to be spherical, monodispersed, and highly stable, with significant negative zeta potential indicating strong electrostatic repulsion. The long-term stability test revealed that PBS at pH 6 and 7 may be good options to maintain AgNPs properties in dispersion for over six months, highlighting the importance of dispersion media in preserving AgNPs functionality. The AgNPs exhibited strong antioxidant activity within multiple in vitro assays, with notably high lipoxygenase inhibition. Additionally, the NPs displayed efficient photocatalytic degradation of synthetic dyes under sunlight, and a capacity to absorb UV-B radiation, indicating promising photoprotective properties. The AgNPs exhibited a cytogenotoxic effect as proved by the two-fold reduction in mitotic index, with the effect being dependent on concentration. The meristematic cells also presented numerous chromosomal aberrations after treatment with AgNPs. These nanoparticles can be further researched as potential antimitotic agents. Overall, the multifunctionality of *A. melanocarpa*-mediated AgNPs—encompassing stability, bioactivity, and environmental responsiveness—emphasizes their potential as eco-friendly nanomaterials for use in biomedical, cosmetic, and environmental remediation applications. This work contributes to the growing field of phytochemical-based nanotechnology and provides a reproducible platform for the green synthesis of metal nanoparticles.

## Figures and Tables

**Table 1:** Phenolic compounds in extract and supernatant (µg/mL).

Compounds	Extract	Supernatant
Gentisic acid	0.608 ± 0.042	<LOQ
Chlorogenic acid	81.406 ± 0.814	2.289 ± 0.045
4-O-caffeoylquinic acid	22.902 ± 2.061	2.468 ± 0.296
Hyperoside	9.422 ± 0.942	0.611 ± 0.079
Isoquercitrin	8.981 ± 0.449	0.658 ± 0.066
Rutoside	5.607 ± 0.112	1.302 ± 0.156
Quercitrin	<LOQ	ND
Quercetin	1.054 ± 0.063	ND

<LOQ—below the limit of quantification of the analytical method; ND—not detected.

**Table 2:** EC_50_ (μg/mL) values for the *A. melanocarpa* berry extract and derived AgNPs in the antioxidant tests.

Sample/Antioxidant Test	Lipoxygenase Inhibition	DPPH Radical Scavenging	Metal Ion Chelating	Hydroxyl Radical Scavenging
Extract	36.44 ± 3.88	80.45 ± 0.52	438.34 ± 1.97	181.17 ± 0.39
AgNPs	18.29 ± 0.52	224.74 ± 5.27	284.11 ± 1.44	155.05 ± 0.63

**Table 3:** SPF values for *A. melanocarpa* berry extract and its derived AgNPs.

Sample and Concentration (g %)	SPF Value
Extract 0.01	2.09
Extract 0.02	2.84
AgNPs 0.005	2.78
AgNPs 0.01	4.50
AgNPs 0.02	8.24

## Data Availability

Data are contained within the article.

## Abbreviation

AgNPs	silver nanoparticles
AgNO_3_	silver nitrate
LC-MS	liquid chromatography coupled to mass spectrometry
SPF	sun protection factor
UV-Vis	ultraviolet-visible spectroscopy
FTIR	Fourier-transform infrared spectroscopy
PDI	polydispersity index
DLS	dynamic light scattering
SEM	scanning electron microscopy
EDX	energy-dispersive X-ray analysis
TEM	transmission electron microscopy
DPPH	2,2-diphenyl-1-picrylhydrazyl
